# Active inference and oculomotor pursuit: The dynamic causal modelling of eye movements

**DOI:** 10.1016/j.jneumeth.2015.01.003

**Published:** 2015-03-15

**Authors:** Rick A. Adams, Eduardo Aponte, Louise Marshall, Karl J. Friston

**Affiliations:** a The Wellcome Trust Centre for Neuroimaging, Institute of Neurology, University College London, 12 Queen Square, London WC1N 3BG, UK; b Translational Neuromodeling Unit (TNU), Institute for Biomedical Engineering, University of Zurich & ETH Zurich, Wilfriedstr. 6, 8032 Zurich, Switzerland; c Sobell Department of Motor Neuroscience and Movement Disorders, UCL Institute of Neurology, 33 Queen Square, London WC1N 3BG, UK

**Keywords:** Oculomotor control, Pursuit, Active inference, Dynamic causal modelling, Precision

## Abstract

**Background:**

This paper introduces a new paradigm that allows one to quantify the Bayesian beliefs evidenced by subjects during oculomotor pursuit. Subjects’ eye tracking responses to a partially occluded sinusoidal target were recorded non-invasively and averaged. These response averages were then analysed using dynamic causal modelling (DCM). In DCM, observed responses are modelled using biologically plausible generative or forward models – usually biophysical models of neuronal activity.

**New method:**

Our key innovation is to use a generative model based on a normative (Bayes-optimal) model of active inference to model oculomotor pursuit in terms of subjects’ beliefs about how visual targets move and how their oculomotor system responds. Our aim here is to establish the face validity of the approach, by manipulating the content and precision of sensory information – and examining the ensuing changes in the subjects’ implicit beliefs. These beliefs are inferred from their eye movements using the normative model.

**Results:**

We show that on average, subjects respond to an increase in the ‘noise’ of target motion by increasing sensory precision in their models of the target trajectory. In other words, they attend more to the sensory attributes of a noisier stimulus. Conversely, subjects only change kinetic parameters in their model but not precision, in response to increased target speed.

**Conclusions:**

Using this technique one can estimate the precisions of subjects’ hierarchical Bayesian beliefs about target motion. We hope to apply this paradigm to subjects with schizophrenia, whose pursuit abnormalities may result from the abnormal encoding of precision.

## Highlights

• We use a normative (Bayes optimal) model of oculomotor pursuit.
• We average the empirical responses of subjects performing a pursuit paradigm.
• We invert these responses using the pursuit model and dynamic causal modelling.
• We thereby estimate the precision of subjects’ Bayesian beliefs from their pursuit.
• This could be used to quantify abnormal precision encoding in schizophrenia.

## 1 Introduction

This paper considers the modelling of oculomotor pursuit using active inference – a normative or Bayes-optimal formulation of action and perception which has been used to address a range of issues in the cognitive neurosciences (Friston et al., 2010a). In a previous paper, we formulated oculomotor control during smooth pursuit eye movements (SPEM) in terms of active inference, with a special focus on how representations of uncertainty or precision could affect eye tracking behaviour (Adams et al., 2012). We established that impairment in the encoding of precision (inverse variance of random fluctuations) at higher levels of a hierarchical model of oculomotor control (e.g., frontal eye fields or prefrontal cortex) resulted in several SPEM abnormalities characteristic of schizophrenia; e.g., a greater slowing of pursuit during target occlusion. In this work, we use a similar generative model to predict empirical eye movements, and thereby make inferences about how subjects optimise their oculomotor responses to moving targets. In particular, we were interested in whether we could induce changes in the precision subjects ascribe to sensory information (by changing the precision of target motion) and infer these subjective changes from measured eye movements.

The model of pursuit used below is based upon active inference. Active inference is a corollary of the free energy principle – a normative model of behaviour that appeals to Bayes optimality principles. In brief, the principle says that we sample sensory inputs to minimise prediction errors. Clearly, prediction errors depend upon predictions and inference about hidden states of the world causing sensory data. A crucial aspect of this inference is the proper weighting of sensory evidence and prior beliefs. Operationally, this rests upon weighting prediction errors in accord with their precision (reliability or inverse variability). This is formally identical to weighted least squares in statistics. Anecdotally, one can regard prediction errors as reporting what is newsworthy (what cannot be predicted) and precision turns up the ‘volume’ of processing channels with more reliable news.

In this paper, we present the methodology that enables one to quantify subjective precision on the basis of empirical eye movements – as a prelude to comparing normal and schizophrenic cohorts (see Section 3). If changes in subjective precision due to alterations in stimulus attributes can be estimated from pursuit data, then perhaps abnormalities of cortical precision found in psychiatric illness can be disclosed.

This paper comprises the following sections. Section 2.1 provides a brief introduction to active inference and predictive coding. Active inference provides a normative model of oculomotor behaviour, given a generative model that subjects used to predict their behaviour, described in Section 2.2. Section 2.3 provides a brief overview of dynamic causal modelling – a standard variational Bayesian scheme for inverting dynamic or state space models. Section 2.4 describes the experimental paradigm used to elicit oculomotor pursuit under visual occlusion and Section 3 presents the dynamic causal modelling results using the active inference model. Section 4 concludes with some comments about the potential applications of this non-invasive approach to quantifying subjective beliefs or expectations entertained by subjects – and how the scheme can be extended to cover neurophysiological responses.

## 2 Materials and methods

### 2.1 Active inference, generalised filtering and free energy

This section introduces active inference in terms of generalised Bayesian filtering – also known as predictive coding. In brief, active inference can be regarded as equipping standard Bayesian update schemes with classical reflex arcs that enable action to fulfil predictions about (hidden) states of the world. We will describe the formalism of active inference in terms of differential equations describing the dynamics of the world – and internal states of the visual–oculomotor system. This scheme is used in subsequent sections to predict pursuit movements under different levels of confidence (precision) about hierarchical predictions.

Active inference is based on three assumptions that formalise the notion that the brain generates predictions of its sensory samples to confirm hypotheses about the state of the world – and how the world is sampled:

• The brain minimises the free energy of sensory inputs defined by a generative model.
• The generative model used by the brain is hierarchical, nonlinear and dynamic.
• Neuronal firing rates encode the expected state of the world, under this model.

The first assumption is the free energy principle, which leads to active inference in the embodied context of action. The free energy here is a proxy for Bayesian model evidence. In Bayesian terms, minimising free energy means that the brain maximises the evidence for its model of sensory inputs (Gregory, 1980; Ballard et al., 1983; Dayan et al., 1995; Olshausen and Field, 1996; Grossberg et al., 1997; Bialek et al., 2001; Knill and Pouget, 2004), in accord with the *Bayesian brain* hypothesis (Yuille and Kersten, 2006; Maloney and Zhang, 2010). If we also allow action to maximise model evidence we get *active inference* (Friston et al., 2010a). In this setting, desired movements are specified in terms of prior beliefs about hidden states in the generative model. Action then realises prior beliefs by sampling sensory inputs to provide evidence for those expectations. The second assumption above is motivated by noting that the world is both dynamic and nonlinear and that hierarchical structure emerges inevitably from a separation of temporal scales (Ginzburg, 1955; Haken, 1983). The final assumption is the Laplace assumption that, in terms of neural codes, leads to the *Laplace code*, which is arguably the simplest and most flexible of all candidate codes (Friston, 2009).

Under these assumptions, action and perception can be regarded as the solutions to coupled differential equations describing the dynamics of the real world, action and perception (Friston et al., 2010a):

(1) $$\begin{array}{l} s=\text{g}(\text{x},\text{v},a)+\omega_{s}\\ \dot{\text{x}}=\text{f}(\text{x},\text{v},a)+\omega_{x} \end{array}$$

(2) $$\begin{array}{l} \dot{a}=-\partial_{a}F(\tilde{s},\tilde{\mu })\\ \dot{\tilde{\mu }}=D\tilde{\mu }-\partial_{\tilde{\mu }}F(\tilde{s},\tilde{\mu }) \end{array}$$

**Fig. 1:**
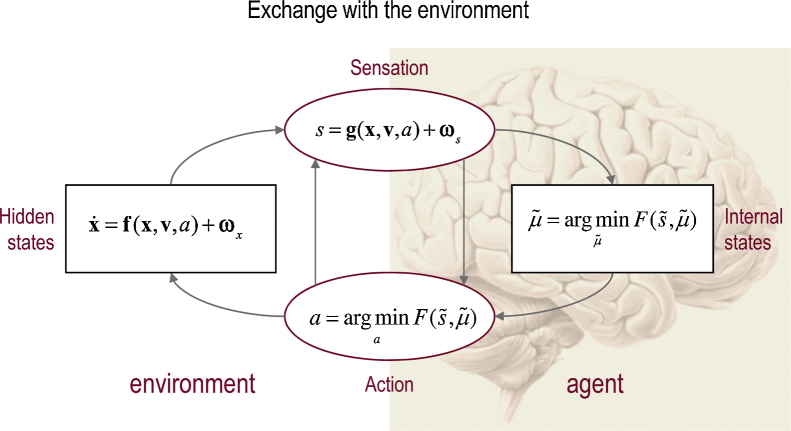
Exchange with the environment. This schematic illustrates the dependencies among various quantities modelling exchanges of an agent with the environment. It shows the states of the environment and the system in terms of a probabilistic dependency graph, where connections denote directed dependencies. The quantities are described within the nodes of this graph – with exemplar forms for their dependencies on other variables (see main text). Hidden and internal states of the agent are separated by action and sensory states. Both action and internal states – encoding posterior or conditional expectations about hidden states – minimise free energy. Note that hidden states in the real world and the form of their dynamics can be different from that assumed by the generative model; this is why hidden states are in bold and internal states are in italics. See main text for further details.

See Fig. 1 for a schematic summary of the conditional dependencies implied by Eqs. (1) and (2). For clarity, real-world states are written in boldface, while the states of the agent are in italics. The ∼ notation denotes variables in generalised coordinates of motion where $\tilde{s}=(s,s^{\prime },s^{\prime \prime },\ldots )$ (Friston et al., 2010b). The pairs of equations are coupled because sensory states *s*(*t*) depend upon action *a*(*t*) through non-linear functions (**g**, **f**) of hidden states and causes (**x**, **v**), while action depends upon sensory states through internal states $\tilde{\mu }(t)$. Internal states play the role of expectations about hidden states that minimise free energy –

(3) $$F=E_{q}[\text{ln}\;q(\tilde{x},\tilde{v}|\tilde{\mu })-\text{ln}\;p(\tilde{s},\tilde{x},\tilde{v}|m)]$$

– or maximise (a lower bound on) Bayesian model evidence $\ln p(\tilde{s}|m)\geq -F$. Here, $q(\tilde{x},\tilde{v}|\tilde{\mu })$ is an approximate posterior density over hidden variables $\left(\tilde{x},\tilde{v}\right)$ that is parameterised by their expected values. Note that the free energy depends upon a generative model:

(4) $$p(\tilde{s},\tilde{x},\tilde{v}|m)=p(\tilde{s}|\tilde{x},\tilde{v})p(\tilde{x},\tilde{v}|m)$$

This model, denoted by *m*, is usually specified in terms of a likelihood and prior (see below). Hidden causes can be thought of as inputs or perturbations to hidden states that produce sensations. In this paper, the hidden cause is a force on a target and the hidden states are the ensuing motion of the target (and eye). Eq. (1) describes the dynamics of hidden states and causes in the world and how these generate sensory data. These equations are stochastic because sensory states and the motion of hidden states are subject to random fluctuations $\omega_{s},\omega_{x}$. The second pair of differential equations (Eq. (2)) corresponds to *action* and *perception* – they constitute a gradient descent on variational free energy. The differential operator D returns the generalised motion of the conditional expectations – such that $D\tilde{\mu }=(\mu^{\prime },\mu^{\prime \prime },\mu^{\prime \prime \prime },\ldots )$.

To perform simulations using this scheme, one simply integrates or solves Eqs. (1) and (2) to simulate (neuronal) dynamics that encode expectations and ensuing action. The vector $D\tilde{\mu }$ is handled numerically by truncating the order of generalised motion to a small number (usually between two and six). One can do this because the precision of high order motion disappears quickly, even for relatively smooth fluctuations. The variational free energy depends upon a generative model, which we assume has the hierarchical form shown in Eq. (5), in which the hierarchical level is denoted by (*i*):

(5) $$\begin{array}{l} s=g^{\left(1\right)}(x^{\left(1\right)},v^{\left(1\right)})+\omega_{v}^{\left(1\right)}\\ {\dot{x}}^{\left(1\right)}=f^{\left(1\right)}(x^{\left(1\right)},v^{\left(1\right)})+\omega_{x}^{\left(1\right)}\\ \vdots \\ v^{\left(i-1\right)}=g^{\left(i\right)}(x^{\left(i\right)},v^{\left(i\right)})+\omega_{v}^{\left(i\right)}\\ {\dot{x}}^{\left(i\right)}=f^{\left(i\right)}(x^{\left(i\right)},v^{\left(i\right)})+\omega_{x}^{\left(i\right)}\\ \vdots \end{array}$$

This equation denotes a generative model *m* that specifies a probability density function over sensory inputs and hidden states and causes (Eq. (4)). This probability density is needed to define the free energy (Eq. (3)) and rests on Gaussian assumptions about random fluctuations $\left(\omega_{x}^{\left(i\right)},\omega_{v}^{\left(i\right)}\right)$ on the motion of hidden states and causes. These fluctuations play the role of sensory noise at the first level and induce uncertainty about states at higher levels. The (inverse) amplitudes of these fluctuations are quantified by their precisions $\left(\Pi_{x}^{\left(i\right)},\Pi_{v}^{\left(i\right)}\right)$.

The deterministic part of the model is specified by nonlinear functions $\left(g^{\left(i\right)},f^{\left(i\right)}\right)$ of hidden states and causes that generate dynamics and sensory consequences. Hidden causes link hierarchical levels, whereas hidden states link dynamics over time. Hidden states and causes are abstract quantities that the brain uses to explain or predict sensations – like the motion of an object in the field of view. In hierarchical models of this sort, the output of one level acts as an input to the next; at the bottom of the model is not $v^{\left(0\right)}$ but *s*, the sensations it is trying to predict. This input can produce complicated convolutions with deep (hierarchical) structure, as we will see examples of this later.

In terms of the biological implementation of active inference, expectations can be updated using predictive coding (Rao and Ballard, 1999; Friston, 2005), which minimises free energy in the form of prediction errors. In other implementations of the active inference framework, action is produced by proprioceptive predictions that descend to the level of (pontine) cranial nerve nuclei and the spinal-cord. These engage classical reflex arcs to suppress proprioceptive prediction errors and elicit the predicted motor trajectory. The reduction of action to classical reflexes follows because the only way that action can minimise free energy is to change sensory (proprioceptive) prediction error. In short, active inference can be regarded as equipping a generalised predictive coding scheme with classical reflex arcs: see Adams et al. (2013a), Friston et al. (2010a) for details.

Active inference in the oculomotor system may eschew an explicit computation of proprioceptive prediction errors in cranial nerve nuclei – because the oculomotor system does not have to contend with context-sensitive loads on the eye (and has to produce rapid movements). Indeed, proprioceptive deafferentation does not affect oculomotor function in monkeys (Lewis et al., 2001). This suggests proprioceptive predictions must be transformed into motor commands by a simple inverse model, rather than being realised by a classical reflex. This inverse model is simple because proprioceptive predictions and motor commands are in the same (motor) frame of reference. Interestingly, the inclusion of oculomotor delays in the current model mandates a simple inverse modelling of delayed kinematics (Perrinet et al., 2014). Having said this, the (peripheral) mechanism by which descending proprioceptive predictions produce oculomotor commands is not important for our purposes: we are interested in the effects – and estimation – of precision in the (central) model.

In summary, we have derived the dynamics of perception and action using a free energy formulation of Bayes-optimal exchanges with the world and a generative model that can be implemented a biologically plausible fashion. A technical treatment of the material above is found in Friston et al. (2010), which provides the details of the generalised filtering used in subsequent sections. To use this scheme in any particular setting, one has to specify the particular generative model in Eq. (5). We now turn to the oculomotor pursuit model used in this work.

### 2.2 Oculomotor pursuit model

The oculomotor pursuit model used here is based on the generative model of SPEM described in Adams et al. (2012) but with one fundamental change. Our previous model was of smooth pursuit only – while it could generate catch-up movements of saccade-like speed, it could not generate *anticipatory* movements. In this application, we wanted to model grand averaged empirical eye traces, and so we had to choose between removing saccades to create average eye velocity traces and averaging eye displacements *in toto*. The second option was preferred, because removing the saccadic portion of the trace would dismiss the synergy between saccades and SPEM during target occlusion (Orban de Xivry et al., 2006), and suppress the anticipatory nature of the eye movements we observed.

This oculomotor pursuit model is essentially a model designed to generate ‘grand averaged’ pursuit movements (which include both SPEM and saccades): it does not reproduce the enormous variability of single trial data. It is neither a model of smooth pursuit per se, nor of separate (pursuit and saccadic) systems: it is not designed to explain how pursuit and saccades might operate in isolation, unlike many models of oculomotor control. *The purpose of this model is to derive estimates of subjective precision at different levels in a hierarchical model of pursuit*: these precisions are key parameters, which – if the brain performs Bayesian inference–must exist ‘in the head’. In future work, we will correlate estimates of subjective precision (from grand averaged pursuit data) with neuronal estimates of synaptic gain (from grand averaged MEG data) that are thought to encode precision. If successful, this will serve as a validation of inverting models of average pursuit trajectories to provide a non-invasive assay of synaptic gain (i.e., subjective precision) in different subject cohorts. The remaining parameters (and model structure) are not intended to be biologically realistic, except in the general sense of realising a hierarchical Bayesian model of a smoothly moving target.

Eye movements are modelled as if they were driven reflexively by descending predictions based upon the following beliefs: the subject believes there is an invisible location – moving sinusoidally along a horizontal line–that is attracting a target. Crucially, the subject also believes that the centre of gaze is attracted to this invisible location, the target or both. This means that the eye movements do not always try to track the target itself, but sometimes a point which is always just ahead of the target. The latter is useful when a target is moving quickly; for example, as when ice hockey coaches advise young players not to try to hit the puck itself, but ‘where the puck is going’. Furthermore, attraction to the invisible location and target may or may not depend upon whether they lie behind an occluder. This enables the model to make anticipatory eye movements; for example, if the target is occluded the eye can track the invisible attracting location instead. The relative attraction of the invisible location and target – and the influence of the occlude – depends upon the (kinetic) parameters of each subject's generative model (see below). This model allows for many contingencies and entails a relatively large number of parameters. However, we will see later that redundant parameters (or model components) can be eliminated using Bayesian model optimisation.

**Fig. 2:**
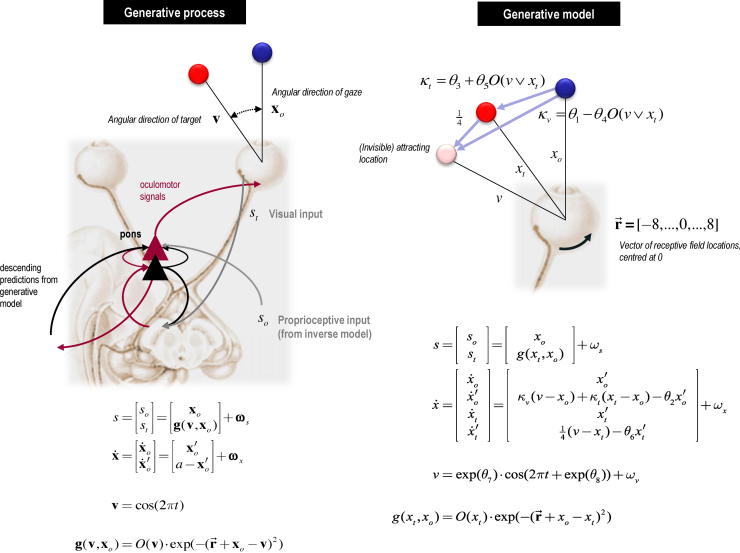
Generative process and model of oculomotor pursuit movements. This schematic illustrates the process (left panel) and generative model of that process (right panel) used to simulate Bayes optimal pursuit. The graphics on the left show a putative predictive coding scheme (with superficial pyramidal cells in red and deep pyramidal cells in black in the pontine nuclei) processing proprioceptive information during oculomotor pursuit. These cells receive proprioceptive information from an inverse model in the subcortical oculomotor system and respond reflexively to minimise proprioceptive prediction error through action. This prediction error rests on descending predictions from the generative model on the right. The actual movement of the target is determined by a hidden cause (target location), which determines the visual input for any given direction of gaze. The generative model entails beliefs about how the target and eyes move. In brief, this model includes an invisible location that attracts the target, causing it to move. Crucially, the agent believes that its centre of gaze is attracted to this location (and the target), where the forces of attraction may (or may not) depend upon occlusion of the target and its attracting location. These forces of attraction are illustrated with lilac arrows in the top right; the arrows are labelled with their respective multipliers from the equations directly below. Please see main text for a description of the variables in the equations describing the motion of hidden states and how they depend upon hidden causes. Note that real states that are hidden from observation in the real world are in bold, whereas the hidden states assumed by the generative model are in italics. (For interpretation of the references to color in the text, the reader is referred to the web version of this article.)

We now consider the model in more detail (also see Fig. 2). The ‘real world’ generating sensory inputs is shown on the left of Fig. 2. We go through the equations in turn:

(6) $$s=\left[\begin{array}{c} s_{o}\\ s_{t} \end{array}\right]=\left[\begin{array}{c} {\text{x}}_{o}\\ \text{g}(\text{v},{\text{x}}_{o}) \end{array}\right]+\omega_{s}$$

Eq. (6) says that the world provides sensory input in two modalities. First, the output of an oculomotor inverse model reports the (horizontal) angular displacement of the eye *s*_0_ and corresponds to the centre of gaze in extrinsic coordinates **x**_0_. Second, visual input reports the angular position of a target in a retinal (intrinsic) frame of reference *s*_t_. This input models the response of visual channels, each equipped with a Gaussian receptive field with a width of one angular unit and deployed at intervals of one angular unit (about 2° of visual angle). These receptive fields are centred on the locations in the vector $\vec{\text{r}}=[-8,\ldots ,0,\ldots ,8]$, where 0 is the centre of gaze. Crucially, this visual input can be occluded by a function of target location O(v)∈[0,1]:

(7) $$\text{g}(\text{v},{\text{x}}_{o})=O(\text{v})\cdot \text{exp}(-{\left(\vec{\text{r}}+{\text{x}}_{o}-\text{v}\right)}^{2})$$

(8) v=cos(2πt)

This means that whenever the sinusoidally-varying target location (hidden cause) **v** is behind the occluder, visual input falls to zero. The response of each visual channel depends upon the distance of the target from the centre of gaze. This is just the difference between the oculomotor angle and target location in an extrinsic frame of reference: **x**_o_–**v**.

(9) $$\dot{\text{x}}=\left[\begin{array}{c} {\dot{\text{x}}}_{o}\\ {{\dot{\text{x}}}^{\prime }}_{o} \end{array}\right]=\left[\begin{array}{c} {{\text{x}}^{\prime }}_{o}\\ a-{{\text{x}}^{\prime }}_{o} \end{array}\right]+\omega_{x}$$

Eq. (9) describes the hidden states of this model which comprise oculomotor angle and velocity $\left({\text{x}}_{o},{{\text{x}}^{\prime }}_{o}\right)$, where velocity is driven by action and decays to zero, with a time constant of one time step (about 16 ms). This means the action applies forces to the oculomotor plant, which responds with a degree of viscosity.

The generative model is shown on the right of Fig. 2 and detailed below. It has a similar form – at the sensory level the models are identical (compare Eqs. (10) and (6), and (11) and (7)), although the subject's estimation of target position *x*_t_ has replaced its real world value **v**:

(10) $$s=\left[\begin{array}{c} s_{o}\\ s_{t} \end{array}\right]=\left[\begin{array}{c} x_{o}\\ g(x_{t},x_{o}) \end{array}\right]+\omega_{s}$$

(11) $$g(x_{t},x_{o})=O(x_{t})\cdot \text{exp}(-{\left(\vec{\text{r}}+x_{o}-x_{t}\right)}^{2})$$

Note that the sensory input is exactly the same as the sensations generated by the real-world process (Eq. (6)). However, there are two important differences between the generative process and the generative model of the process: there is no action and both the target and centre of gaze are drawn to a (fictive) attracting location whose position is encoded by a hidden cause *v*:

(12) $$v=\text{exp}(\theta_{7})\cdot \text{cos}(2\pi t+\text{exp}(\theta_{8}))+\omega_{v}$$

Eq. (12) shows the attracting location *v* is a sinusoidal function of time with parameters controlling its amplitude and phase $\left(\theta_{7},\theta_{8}\right)$. Further parameters control the evolution of hidden states (note that the expected motion ${x^{\prime }}_{o}$ is distinct from the motion of the expectation ${\dot{x}}_{o}$: heuristically, this is like the difference between motion-sensitive responses in V5 and the motion of peak responses in V1):

(13) $$\dot{x}=\left[\begin{array}{c} {\dot{x}}_{o}\\ {{\dot{x}}^{\prime }}_{o}\\ {\dot{x}}_{t}\\ {{\dot{x}}^{\prime }}_{t} \end{array}\right]=\left[\begin{array}{c} {x^{\prime }}_{o}\\ \kappa_{v}(v-x_{o})+\kappa_{t}(x_{t}-x_{o})-\theta_{2}{x^{\prime }}_{o}\\ {x^{\prime }}_{t}\\ \frac{1}{4}(v-x_{t})-\theta_{6}{x^{\prime }}_{t} \end{array}\right]+\omega_{x}$$

The hidden location v attracts the target – i.e. changes in target velocity ${{\dot{x}}^{\prime }}_{t}$ are driven by the distance between the target and invisible location $\left(v-x_{t}\right)$ – but with a viscosity encoded by $\theta_{6}$. The viscosity of eye movements is encoded by $\theta_{2}$. Changes in eye velocity ${{\dot{x}}^{\prime }}_{o}$ are determined by a weighted combination of the distances between the eye and the invisible location and target $\kappa_{v}(v-x_{o})+\kappa_{t}(x_{t}-x_{o})$. The relative strength of these two forces depends on whether the target or invisible locations are occluded:

(14) $$\kappa_{v}=\theta_{1}-\theta_{4}O(v\vee x_{t})$$

(15) $$\kappa_{t}=\theta_{3}+\theta_{5}O(v\vee x_{t})$$

Each strength $\left(\kappa_{v},\kappa_{t}\right)$ has a fixed component and an occluder-dependent component (Eqs. (14) and (15)) that depends on the remaining kinetic parameters $\left(\theta_{1},\theta_{3},\theta_{4},\theta_{5}\right)$. Here, the occluder is a function of the disjunction (inclusive ‘or’) of attractor and target location – such that changes in κ anticipate the emergence of $x_{t}$ from the occluder).

The resulting set up is shown on the upper right of Fig. 2: the generative model believes that the centre of gaze (blue circle) is attracted to the hidden location or cause (pink circle) and the target (red circle). The hidden location drives eye movement when the target is either visible or occluded. The priors for the parameters are chosen such that when the occluder is present, the strength of attraction to the hidden location increases and the strength of attraction to the target decreases, as one might expect. Finally, the model parameters include the precision of random fluctuations at each level (Eqs. (10), (12) and (13)); namely, the sensory input $\left(\omega_{s}\right)$, the motion of the hidden states $\left(\omega_{x}\right)$ and the hidden cause $\left(\omega_{v}\right)$. The ω terms are independent random effects.

**Fig. 3:**
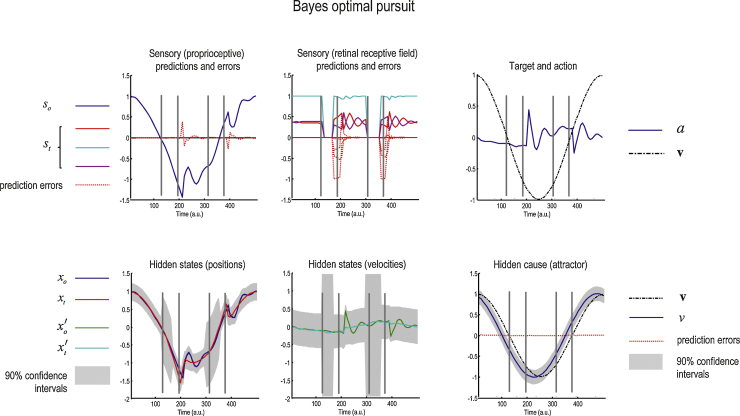
Simulation of pursuit of a partially occluded target. This figure reports the posterior or conditional expectations about hidden states and causes during the simulation of oculomotor pursuit movements over one cycle of target motion. The position of the occluder is illustrated in all panels by the parallel grey lines – these do not always coincide with changes in state estimates because the latter are calculated in 16 ms time steps. The upper left panel shows the proprioceptive predictions (blue line) and prediction errors (dotted red lines). The upper middle panel shows the predicted retinal input – the red, cyan and purple lines correspond to the middle three of an array of 17 photoreceptors (the target is centrally fixated) – and the dotted red lines are prediction errors. The sensory predictions are based upon the expectations of hidden oculomotor (blue line) and target (red line) angular displacements shown on the lower left; the corresponding velocities are shown as the green (eye) and cyan (target) lines on the lower middle graph. The grey regions correspond to 90% Bayesian confidence intervals. Note the increase in uncertainty about the location of the target during periods of occlusion. The hidden cause of these displacements (broken black line) is shown with its conditional expectation (blue line) in the lower right panel. The true cause and action are shown on the upper right. The action (blue line) is responsible for oculomotor displacements and is driven by proprioceptive prediction errors.

Having specified the generative process and model, we can now solve the active inference scheme in Eqs. (1) and (2) and use this to predict observed behaviour. Fig. 3 shows the posterior or conditional expectations about hidden states and causes during the simulation of pursuit over one cycle of target motion. This simulation assumes some particular values for the parameters that we will use as prior expectations later (see Table 1). In both the simulations and later empirical studies the target was occluded whenever it passed behind an occluder at a leftward displacement of 0–0.8 of maximal target displacement. In this simulation, the expected log precision of the random fluctuations of sensory input, motion of hidden states and the hidden cause in the generative model were all set to four. This corresponds to an expected standard deviation of exp(−2)=0.135 (of maximum target displacement). We suppressed the random fluctuations in the generative process so that the target motion was infinitely precise. (In our subsequent experiment, random fluctuations in the generative process were either suppressed or accentuated, and the estimated or subjective precision was inferred from their eye movements.)

The upper left and middle panels of Fig. 3 show the predicted sensory input (coloured lines) and sensory prediction errors (dotted red lines). In the upper middle graph, the red, cyan and purple lines correspond to photoreceptor activity over an array of 17 sensory inputs: only the middle three inputs show activity because the target is well-fixated. In the upper left graph, the proprioceptive predictions (blue lines) reflect veridical pursuit; even during occlusion when visual input disappears. These sensory predictions are based upon the expectations of hidden oculomotor (blue line) and target (red line) angular displacements shown on the lower left. In the lower middle graph, the green (oculomotor) and cyan (target) lines are the corresponding velocities. The grey regions correspond to 90% Bayesian confidence intervals. Note the increase in uncertainty about the location of the target during periods of occlusion. The hidden cause of these displacements (broken black line) is shown with its conditional expectation (blue line) in the lower right panel. The true cause and action are shown on the upper right. The action (blue line) is responsible for oculomotor displacements and is driven by proprioceptive prediction errors (red lines in the upper left panel). This dependency of action on proprioceptive prediction errors effectively closes the action perception loop.

**Fig. 4:**
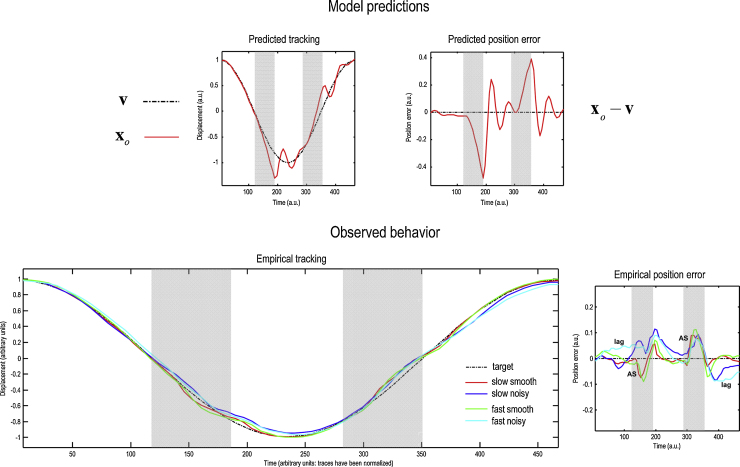
Simulated and empirical tracking. The target pursuit produced by the action simulated in the previous figure is shown in the upper panels. The upper left panel shows the trajectory of the target (broken black line) and the centre of gaze (red line). The difference between these angular displacements (eye position–target position) is the pursuit position error on the upper right. The presence of the occluder is indicated by the grey blocks. The grand averaged and normalised empirical trajectories and position errors for all four conditions are shown in the lower left and right panels respectively. Note that the position error reverses sign halfway through the sinusoid; i.e., the eye is always ahead of the target during occlusion in the ‘smooth’ condition (red and green lines). ‘AS’ denotes its anticipatory saccadic movement, and ‘lag’ its lag behind the target.

The ensuing target trajectory and pursuit is shown in Fig. 4 (upper row). The upper left panel shows the trajectory of the target (broken black line) and the centre of gaze (red line). The difference between these angular displacements is the position error on the upper right. The values of the parameters in Table 1 were chosen to produce movements that caricature normal pursuit. Examples of real trajectories and position errors are shown in the lower left and right panels respectively (normalised with respect to time and displacement). These are the averaged responses under different experimental conditions that will be analysed later. Both the simulated and empirical pursuit trajectories show a deviation from the true target trajectory after it passes behind an occluder (the vertical broken lines), which is corrected when the target re-emerges. This correction generally produces an overshoot.

These simulations reproduce Bayes-optimal (grand averaged) eye movements, given a smoothly moving target trajectory. This Bayesian optimality rests upon the particular generative model used for active inference and its parameters that encode beliefs about how targets move – and induce eye movements. We are now in a position to use this model to generate predictions of subject behaviour and optimise the model parameters. These parameters fall into three sets (see Table 1): the parameters of visual kinetics; the expected log precisions and prior beliefs about the invisible attracting location trajectory.

Our primary focus in what follows is on the expected log precisions and how they are affected by experimental context. Note that we are not determining how the precision parameters ought to change in response to changes in target characteristics: we are estimating how they actually change. This is important because changes in hierarchical precision in a generative model of noisy target motion may not just *reflect* but also *compensate* for changes in the precision of the target. We now turn to the nature of this estimation using dynamic causal modelling.

### 2.3 Dynamic causal modelling of eye movements

In this section, we briefly review the concept of ‘meta-Bayesian’ modelling, model inversion using dynamic causal modelling (DCM) and DCM's application to eye movements. The modelling in this study can be regarded as ‘meta-Bayesian’ because we are using Bayes’ rule twice. First, we assume that our subjects are engaging in active Bayesian inference using a generative model of their sensations $p(s|\theta_{s},m_{s})$, with parameters $\theta_{s}$ (including hidden states and causes) of their (subjective) model *m*_*s*_:

(16) $$p(s|\theta_{s},m_{s})=N(G_{s}(\theta_{s}),\Sigma (\theta_{s}))$$

*G*_*s*_ denotes the non-linear mapping from the model parameters to sensory input, which is subject to Gaussian noise. Given the sensory data they observe (Eq. (16)) we can emulate the Bayesian updates to their beliefs (Eq. (17)):

(17) $$p(\theta_{s}|s,m_{s})=\frac{p(s|\theta_{s},m_{s})p(\theta_{s}|m_{s})}{p(s|m_{s})}$$

We can also emulate their Bayes-optimal action a^*^ which maximises model evidence:

(18) $$a^{*}=\underset{a}{\arg\max}p(s|m_{s})$$

This *subjective model* (Eqs. (16)–(18)) is then absorbed into an *objective model* $m_{o}$ of their behaviour (Daunizeau et al., 2010), illustrated formally in Eqs. (19)–(21):

(19) $$p(a|\theta_{o},\theta_{s},m_{s},m_{o})=N(G_{o}(\theta_{o},a^{*}(\theta_{s})),\Sigma (\theta_{o}))$$

(20) $$p(\theta_{o},\theta_{s}|a,m_{s},m_{o})=\frac{p(a|\theta_{o},\theta_{s},m_{s},m_{o})p(\theta_{o},\theta_{s}|m_{s},m_{o})}{p(a|m_{s},m_{o})}$$

(21) $$\theta_{s}^{*}=\underset{\theta_{s}}{\arg\max}p(a|m_{s},m_{o})$$

This enables one to estimate the parameters (e.g. the precision) of subjective beliefs given the behaviour observed by the experimenter.

For timeseries data like eye tracking responses, Bayesian model inversion usually calls on some form of dynamic causal modelling: a Bayesian model inversion and selection scheme that uses standard Bayesian (variational) procedures to estimate the parameters of time series models – usually specified in terms of differential equations. These differential equations specify predicted observations and form the basis of a likelihood model. The generative model is completed by specifying prior beliefs about model parameters. In our case, the predicted position error $a={\text{x}}_{o}(\theta_{s})-\text{v}+e$, given some generative model parameters, provides the likelihood of the observed position error (averaged over multiple trials), under the assumption of additive Gaussian noise $e∼N(0,\Sigma (\theta_{o}))$:

(22) $$p(a|\theta_{o},\theta_{s},m_{s},m_{0})=N(a^{*}(\theta_{s}),\Sigma (\theta_{o}))$$

(23) $$a^{*}(\theta_{s})={\text{x}}_{o}(\theta_{s})-\text{v}$$

Eq. (22) is from Eq. (19); it shows that the likelihood of the position error depends on both the Bayes optimal position error predicted by the subjective model and the observation noise. Notice that the observation model $G_{o}=a^{*}(\theta_{s})$ is very simple because the subjects’ behaviour is directly available for observation. Prior beliefs about the parameters $p(\theta_{s}|m_{s})$ then provide a full generative model of observations, which can be inverted (see Eqs. (19)–(21)). Table 1 contains the prior expectations, while the prior variance of the (log scaling of the) parameters was set to one half. Note that these (relatively uninformative) priors are our prior beliefs about the model parameters that encode the subjective beliefs of the (grand averaged) subject.

The inversion scheme used in this application is exactly the same as the scheme used to invert dynamic causal models of fMRI and EEG timeseries (see Friston et al. (2007), Friston et al. (2003), Kiebel et al. (2009) for details). Interestingly, it is based upon the same gradient descent that underlies the active inference scheme of the previous section; however here, the posterior expectations of the parameters $\left(\theta_{o}^{*},\theta_{s}^{*})=\theta^{*}=E[\theta |a,\text{v},m_{s}\right]$ optimise the free energy of observed position errors such that it approximates the log model evidence:

(24) $$F(a,\theta^{*})\approx -\text{ln}\;p(a|\text{v},m_{s})$$

This log-evidence can then be used to compare different models in terms of their likelihood – or to perform Bayesian model averaging. Bayesian model averaging is essentially a way of estimating the parameters that relaxes assumptions about a particular model being the correct model. This is achieved by weighting the value of a particular model's parameters by the likelihood of that model.

In what follows, we will consider a large number of models that do or do not allow for changes in various parameters. We will assess the evidence for (changes in) a particular model parameter in terms of the log-evidence of models that do and do not contain (changes in) that parameter. Finally, we will characterise the effects of experimental manipulations on parameters using the Bayesian model averages over all possible models.

All of the analysis software used in this paper and a sample dataset is available as part of the SPM software (www.fil.ion.ucl.ac.uk/spm), in the SPEM_and_DCM toolbox: the (annotated) demo routine is spm_SEM_demo.m. A more generic meta-Bayesian modelling routine (for eye movements but generalisable to other contexts) is called spm_meta_model.m and can be found in the DEM toolbox. The integration of the active inference scheme and subsequent dynamic causal modelling used a local linearisation scheme (Ozaki, 1992) as implemented in spm_ADEM.m and spm_nlsi_GN.m.

### 2.4 Experimental paradigm

In this section, we describe the experimental paradigm used to generate pursuit movements, whose averages are modelled in terms of active inference. Our intention here was to try to induce – in normal brains – hierarchical changes in subjective precision that we have proposed in schizophrenia (as a result of NMDA-R hypofunction in prefrontal cortex: (Adams et al., 2013b). This putative manipulation of expected or subjective precision rests on exploiting (Bayes) optimal neuronal processing of different stimuli.

In brief, normal subjects pursued a sinusoidal target moving behind a visual occluder under two levels of two experimental factors. The first factor changed the precision of the velocity of the target – by making the sinusoidal motion noisy. The second factor was the speed of the sinusoidal motion. Our initial hypothesis was that decreasing the precision of the velocity would decrease the precision of hidden states and causes Π_*x*_, Π_*v*_, relative to sensory precision Π_*s*_. Conversely, we conjectured that the speed manipulation would induce a change in the kinetic parameters but not the precision parameters. In fact, these hypotheses were rather naive and we obtained some rather surprising results that, in retrospect, we could have anticipated.

We acquired pursuit data from 8 healthy human subjects (mean age 27.1 years, 2 female). All subjects were naïve to ocular pursuit tasks, had normal or corrected-to-normal vision and gave written informed consent. The study was approved by UCL Ethics committee (1825/003). The experimental protocol was written in Matlab, using the Psychophysics and Eyelink Toolbox extensions (Brainard, 1997; Cornelissen et al., 2002) and Cogent 2000, developed by the Cogent 2000 team at the WTCN and ICN, and Cogent Graphics developed by John Romaya.

Each subject sat in an enclosed and darkened room, with their head stabilised using a chin rest and head abutments. The target was displayed on a 41 cm by 30 cm DELL UltraSharp 2408WFPb LCD flat screen monitor, 60 cm from the subject. The target consisted of a black dot (2 mm across) surrounded by a white ring (3.5 mm radial width) moving over a black background. Total target diameter was 9 mm or 0.86° visual angle. Target luminance was 18 cd/m^2^ and background luminance was 0.01 cd/m^2^.

The target moved along a horizontal plane, halfway up the screen over 75% of the screen width (28.8° of visual angle). At the beginning of each trial, the target stimulus appeared at either the left or right end of its path, and remained stationary for 1–3 s (the precise time varied randomly). The target then moved horizontally, its velocity varying sinusoidally. One trial consisted of three full cycles of motion. In each trial, the target was occluded between the midline and the furthest 10% of the path from where the target started; i.e., for 40% (11.5°) of the total path, twice per cycle. The occluder was the same colour as the background.

Two variables were varied independently in a 2 × 2 factorial design: the period of the cycle, and the smoothness of the motion. Two different periods were used, of 4.173 s and 5.1 s, whose maximum velocities were 21°/s and 17.2°/s, and in which the occluded periods lasted 615 ms and 752 ms respectively. We refer to these conditions as ‘Fast’ and ‘Slow’, although compared with most pursuit experiments these maximum velocities are moderate to fast. In the ‘Smooth’ motion condition, the target moved sinusoidally. In the ‘Noisy’ motion condition, a Gaussian random walk of variance σ=exp(−0.5) was added to the phase of the target motion, such that:

(25) $$\begin{array}{l} x(t)=\text{cos}(2\pi f(t+\phi (t)))\\ \phi (t)=\phi (t-1)+\omega (t)\\ \omega (t)∼N(0,\sigma^{2}) \end{array}$$

Here *f* is the target frequency and *t* the time in milliseconds. This created rapid fluctuations around an underlying sinusoidal motion, which had the same period as the Smooth trajectory. The ensuing fluctuations were too fast to be tracked with the eyes, and subjects were instructed to follow the ‘average’ position of the target, rather than the fluctuations themselves. Subjects were explicitly asked to maintain pursuit and not to saccade to the side of the occluder. Note that the observer model (Fig. 2, or Eqs. (10)–(15)) does not contain a model of this stochastic process, because we wish to see whether Noisy motion impacts upon the precision parameters in particular.

The experiment consisted of 12 blocks of 4 trials, such that there were 12 trials (36 cycles) of each of the four conditions. Fast and Slow stimuli were presented in the first and second halves of the experiment. Smooth and Noisy stimuli were presented in pseudorandom order, such that every eight trials contained four of each.

Eye movement data – including horizontal and vertical eye movements and pupil diameter – were collected using an infrared eyetracker (Eyelink 1000, SR Research, Ontario, Canada), sampling at 1000 Hz. The eyetracker was recalibrated using an automated calibration routine after every block of 4 trials; this entailed the presentation of a 5 mm white circular target stimulus at ±14° horizontal, ±10° vertical and 0° of visual angle, until the calibration error was <1°. The stored .edf files were converted into ASCII and imported into Matlab. The pursuit trajectory root mean square errors were calculated for each cycle, and those over 3.8 cm were visually inspected. If there was evidence of either a calibration problem or gross distortion from blinking (or complete failure to track the target) the cycle was discarded (<10% total cycles were discarded for any subject). The Slow condition data from three subjects had to be discarded for technical reasons (archiving problems).

Following the usual procedure in the dynamic causal modelling of ERPs (e.g., Garrido et al., 2008), we used the grand average pursuit trajectories (over cycles and subjects) as (precise) data features that would inform our Bayesian model comparison – in which we hoped to find evidence for condition-specific effects on the encoding of precision. Note that this inference goes much further than simply demonstrating significant differences between conditions (e.g., in relation to intertrial or intersubject variability). The grand averages were normalised so that they corresponded to a single cycle of target motion with unit amplitude: this allows us to compare responses from experimental setups with different screen sizes and distances between the screen and the subject (e.g., in our subsequent MEG experiment). The grand averages were then subject to dynamic causal modelling, allowing all (kinetic, precision and prior) parameters (except viscosity parameters) to change with the two (motion noise and speed) experimental factors. Nonnegative precision and prior parameters were estimated in terms of their log scaling – such that a value of 0 corresponds to a scaling by exp(0) = 1 or no change from the prior expectations in Table 1. In addition to estimating these parameters, we also estimated the changes induced by changing target motion noise or speed.

Notice that in this particular application, we are estimating the parameters that explain the average response to multiple noisy trajectories. This is not the same as the average of the parameters underlying the response to each trajectory. In other words, the parameters of the average response are not the average parameters of the responses because the parameters are a nonlinear function of observed responses. The advantage of using the response average is that we can use a deterministic generative model that does not have to consider random or stochastic fluctuations introduced by noisy target motion (or eye movements).

**Fig. 5:**
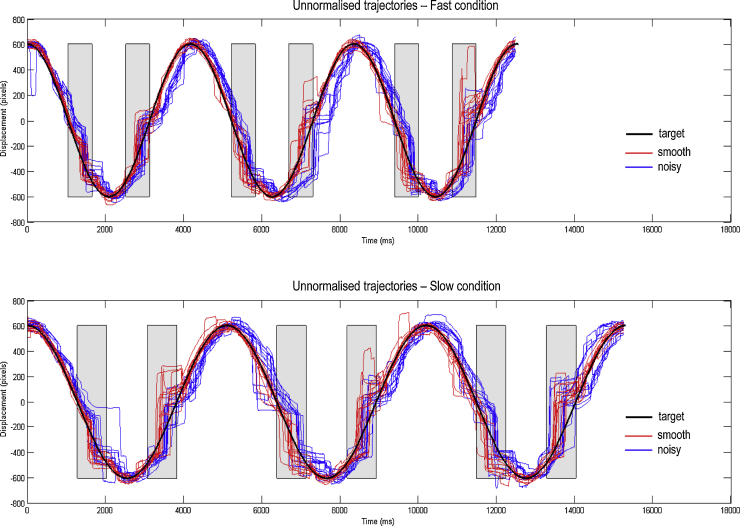
Unnormalised trajectories from a sample subject. This figure illustrates all of the eye trajectories from a single subject, prior to their normalisation and averaging. The upper panels depict the trajectories from the Fast condition and the lower panels those from the Slow condition. The occluders are shown as grey rectangles. It is clear even from this raw data that the eye trajectories in the Noisy condition (blue lines) lag behind those in the Smooth condition (red lines), but the latter track the target (black line) quite well. Note that the target line depicts the actual target position in the Smooth condition, but in the Noisy condition a Gaussian random walk was added to the phase of the target (not shown). (For interpretation of the references to color in this figure legend, the reader is referred to the web version of this article.)

As noted by one of our reviewers, it is possible in theory to compare averaged eye trajectories from the start of the experiment with those from the end, to investigate the timescale over which model parameters are learned. In this paradigm, however, subjects become familiar with the sinusoidal motion and amplitude, occluder position and motion noise within a few trials, and so comparing averages of trials 1–8 and 17–24 may not be the optimal way to assess learning (averaging fewer trials makes parameter estimation difficult as individual trials are quite noisy – see Fig. 5): a different paradigm may be more suitable to characterise learning per se.

## 3 Results and discussion

**Fig. 6:**
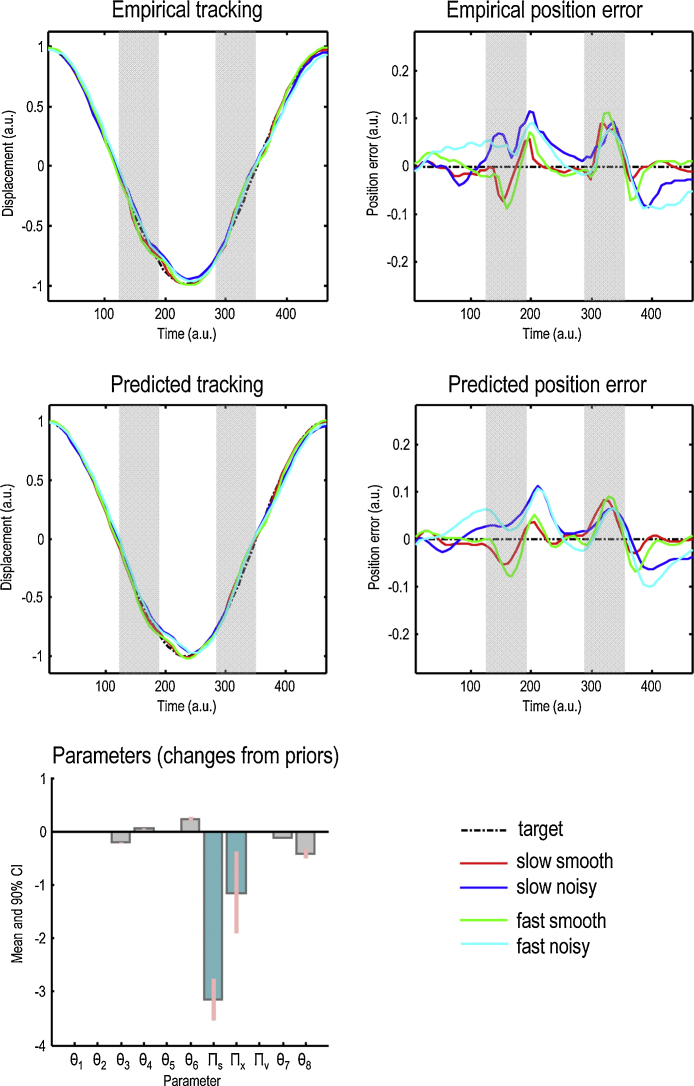
Empirical and predicted tracking. This figure reports the observed and predicted trajectories (upper left panels) and position errors (upper right panels) for the four conditions of our two factor design; namely, Smooth versus Noisy and Slow versus Fast. The predicted (average) responses were based upon the posterior expectations of the parameters of the generative model described in Fig. 2. The lower panel of Fig. 5 shows the posterior expectations of the model parameters (averaged over conditions), plotted as the changes from prior expectations listed in Table 1, and shown in the same order. For clarity, the precision parameters are shown in teal – separating the kinetic (eye movement) parameters from the prior (target movement) parameters. The pink bars correspond to 90% Bayesian confidence intervals.

**Fig. 7:**
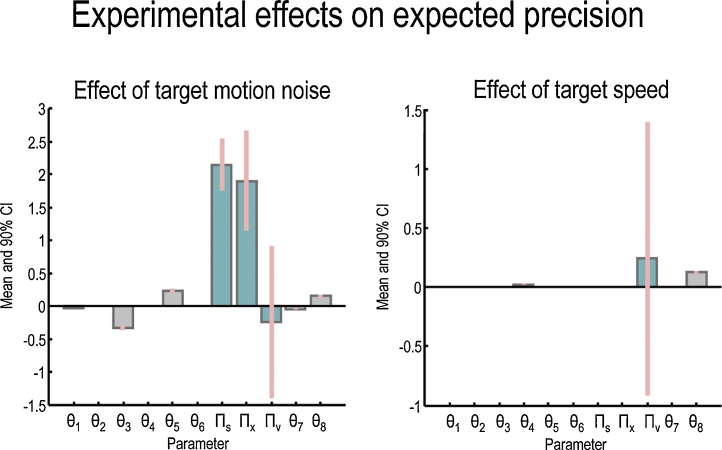
Context dependent parameter changes. These panels show the changes in parameters due to the effect of increasing the noise of target motion (left) and its speed (right). The graphs plot the changes in parameters from baseline with (+) and without (−) changes in noise or speed: the changes in kinetic parameters are absolute, but changes in precision and prior parameters are log scaling factors. Thus moving from Smooth to Noisy conditions increases $\Pi_{s}$ by a factor of $\exp{\left(2.2\right)}^{2}$ – from the left panel. The precision parameters are shown in teal and the pink bars correspond to 90% Bayesian confidence intervals. The confidence intervals around $\Pi_{v}$ are very broad because only large changes in $\Pi_{v}$ have a substantial effect on eye motion. This means there is a lot of uncertainty about its expected value. For reference, absolute values of posterior expectations in a given condition can be determined as follows. The changes in eye kinetics parameters $\left(\theta_{1},\ldots ,\theta_{6}\right)$ from priors to posteriors are absolute; e.g. parameter *θ*_3_ had a prior expectation of 0.5. Its posterior expectation is changed by −0.21 (from Fig. 5, bottom right panel), and target motion noise exerts a further effect on this baseline of ±−0.34 (from Fig. 7, right panel) with no effect of target speed, hence in the Smooth condition $\theta_{3}=0.5-0.21-(-0.34)=0.63$ whereas in the Noisy condition $\theta_{3}=0.5-0.21+(-0.34)=-0.05$. The changes in precision parameters are log scaled, e.g. for the Smooth condition $\Pi_{s}=\text{exp}(4-3.2-2.2)=0.25$ whereas in the Noisy condition, $\Pi_{s}=\text{exp}(4-3.2+2.2)=20$. Finally, changes in the prior beliefs about the attracting location were also log scaled, e.g. in the Fast Noisy condition, $\theta_{8}=(2\pi \text{/}32)\times \text{exp}(-0.4+0.15+0.13)=(2\pi \text{/}32)\times 0.88$.

A sample subject's unnormalised eye trajectories in the Fast and Slow conditions – before averaging – are shown in the upper and lower panels of Fig. 5, respectively. The normalised grand averaged empirical eye trajectories are shown at the bottom left of Fig. 4, together with the position errors (difference between the eye and the target) on the bottom right, for the four conditions of our two factor design; namely, Smooth versus Noisy and Slow versus Fast. Fig. 6 shows the same observed trajectories and position errors (top panels) and the predicted trajectories (middle left panel) and position errors (middle right panel). The predicted responses were based upon the posterior expectations of the parameters after Bayesian model averaging. This averaging used post hoc model optimisation (Friston and Penny, 2011), in which the evidence (marginal likelihood) for many models with reduced numbers of parameters is computed from the posterior density over the parameters of the full model. Free parameters can then be removed from the full model using very precise shrinkage priors. The lower panel of Fig. 6 shows the posterior expectations of the model parameters (averaged over the four conditions). Condition specific changes due to target motion noise and speed are shown in Fig. 7. The parameters are shown in the same order presented in Table 1. For clarity, the log-precision parameters are shown in teal – separating the kinetic parameters from the prior parameters. The pink bars correspond to 90% Bayesian confidence intervals. Note that the precision and prior parameters are log scale parameters.

We first comment on the empirical trajectories. In keeping with previous work on the oculomotor response to the predictable disappearance and reappearance of a target (Bennett and Barnes, 2003; Orban de Xivry et al., 2009), target occlusion causes an anticipatory saccadic movement followed by a loss of eye velocity, seen in the averaged position errors (Fig. 4, lower right panel – ‘AS’) as abrupt advances in eye position ahead of the target shortly after its occlusion begins and before any lag (on average) develops. The only exceptions to this pattern are the traces behind the first occlusion in the Noisy conditions, whose anticipatory movements do not (on average) get ahead of the target because they are smaller, later and occur in the context of greater lag. After the anticipatory saccade, a low velocity is maintained such that when the target reappears the eye is now behind it. Thereafter, the lag is corrected with varying success. The residual pursuit velocities (the averaged eye velocity during the latter half of target occlusion, once saccades of >35°/s were excluded from the data – not shown) were almost identical for both Smooth and Noisy conditions and Fast and Slow speeds: around −3°/s when the eye was decelerating during the first occlusion, and around 5°/s when accelerating during the second occlusion.

We now turn to the condition-specific effects. In brief, the effect of rendering the sensory information imprecise or noisy – the difference between the Smooth (red and green) and Noisy (blue and cyan) trajectories – appears to be greater than the difference between Slow (red and blue) and Fast (green and cyan) pursuits. The effect of making the stimulus noisier is generally to increase the lag of the eye behind the target. This is most marked during the first occlusion (in each cycle) and following the second occlusion – although during the saccade in the second occlusion this effect momentarily disappears. As noted by one of our reviewers, we were using an LCD monitor which is susceptible to motion blur (Elze and Tanner, 2012). In principle, the observed lag in the Noisy condition may be due to a target motion blur; however, contribution of motion blur is probably very small because the biggest lag we observed was during the first occlusion, when no target is visible.

Conversely, the effect of increasing the speed of the target appears to interact with the presence of Noisy motion. Increasing the speed of the Smooth target has little effect, other than slightly increasing the degree to which saccades or subsequent slowing of pursuit overshoot the target before they are corrected. Increasing the speed of the Noisy target does not have this effect, but instead compounds the effect of Noisy motion by increasing the lag of eye behind target, when the latter is visible.

Comparing the empirical and predicted position errors (Fig. 6, middle and top right panels) shows a reasonable, if not perfect, agreement. Most of the deviations from the target trajectory have been reproduced – and, in particular, the quantitative differences induced by changing target motion noise or speed. The bottom panels in Fig. 6 display the posterior expectations of the parameters (averaged over conditions). The point to take from Fig. 6 is that during pursuit of a predictable target, prior precision remains high (ln Π_*v*_ is unchanged at 4) whereas motion and in particular sensory precision are diminished (ln Π_*s*_ drops to around 1). In neurobiological terms, this would mean that in (Bayes) optimal pursuit, subjects are more confident about the underlying period of the target in relation to sensory information than the prior values we used would suggest. In cognitive terms, this means that subjects are attending to the high level (global) Gestalt of motion and less to the local displacement and velocity cues. Having noted this, we now turn to the important results; namely the condition specific changes in these parameters. These are shown in Fig. 7 using the same format as Fig. 6.

The most important point to note is that the biggest effects of target motion noise – by far – are on the precision parameters (Fig. 7 left panel, teal bars) relative to effects on kinetic and prior parameters (grey bars). Between Smooth and Noisy conditions, sensory precision ln Π_*s*_ and state precision ln Π_*x*_ vary by a factor of roughly $\exp{\left(2\right)}^{2}\approx 60$. In comparison, the greatest change in the remaining parameters is a kinetic parameter *θ*_3_, changing by a factor of 14 (see Fig. 7 legend). This is interesting and consistent with our predictions. In other words, the most parsimonious explanation for the effect of changing target precision was, quantitatively, to change the precision or confidence evidenced by subjects in beliefs about the motion of the target and their gaze. Having said this, the pattern of changes in precision – both over experimental conditions and levels of the generative model were not exactly what we predicted.

We had expected that introducing uncertainty into target motion, by making it noisy, would suppress motion and prior precision (the second and third teal bars), and leave sensory precision unchanged (the first teal bar). However, the quantitative results of the dynamic causal modelling suggest something slightly different: it appears that subjects respond to Noisy target trajectories (at either Fast or Slow speeds) by attending more closely to sensory and motion information, while leaving prior precision unchanged. As we predicted, there was a shift in the balance of precision away from upper hierarchical levels (prior precision) and towards lower hierarchical levels (sensory precision), but this came about due to an increase in sensory precision rather than a decrease in prior precision. In retrospect, this is a perfectly optimal response that does not merely reflect but attempts to compensate for the loss of precision about motion in the stimuli.

Here, we are interpreting the balance between sensory and prior precision in terms of attention: in predictive coding, attention can be modelled in a fairly straightforward way through a hierarchical optimisation of expected precision. This reproduces both sensory phenomena – like biased competition – and the psychophysics of the Posner paradigm (see (Feldman and Friston, 2010) for details). The attentional interpretation of precision also echoes the notion of ‘gain control’ in movement being a form of ‘motor attention’ (Lisberger, 2010; Brown et al., 2011). In our context, it appears that subjects respond to noisy stimuli by directing attention to the stimulus (i.e. increasing sensory precision), rather than suppressing confidence in prior beliefs about its motion. This maintenance of prior precision is consistent with the observation that increasing target motion noise had no effect on residual (saccade-free) pursuit velocity (RPV) during occlusion: had prior precision decreased, we would have expected a lower RPV during occlusion, as we have shown in previous modelling work (Adams et al., 2012) – and as is found in many studies of schizophrenic SPEM (O’Driscoll and Callahan, 2008).

As predicted, target speed has no effect on precision parameters: the confidence intervals around the small increase in prior precision span zero (Fig. 7). Instead, the only changes were to the kinetic parameter *θ*_4_ and prior parameter *θ*_8_, suggesting that increasing target speed increased the ‘pull’ of the eye to the attracting location when behind the occluder (i.e. saccades were faster) and increased the phase lag between the target and the attracting location, respectively.

The effects of target motion noise on the kinetic parameters are best expressed in terms of the overall weighting of the eye's attraction to the target $\kappa_{t}$ or attracting location $\kappa_{v}$ (calculated from the other kinetic parameter estimates: Eqs. (14) and (15)). $\kappa_{v}$ always increases during target occlusion–from around 1/6 to 1/4 in each condition – as we expected. We had anticipated that $\kappa_{t}$ would always decrease during target occlusion, but in fact this only occurred during Noisy trials (from around −1/3 to −1/2): the amount of anticipatory saccadic movements during target occlusion in Smooth trials could only be explained by increases in both $\kappa_{v}$ and $\kappa_{t}$ (from around −1/8 to 1/16). Having said this, the kinetic parameters were not our focus – their *raison d’être* was to optimise our estimates of subjective expected precision.

Our next goal will be to show that these precision estimates correlate with measures of neural activity, and thus establish their construct validity. In other words, we hope to use the current DCM paradigm to phenotype behaviour in terms of subjective precision in a normative setting and then show that this behavioural phenotype has neuronal correlates (see below). This involves expressing free energy in terms of prediction errors and then associating predictions and prediction errors with various neuronal populations in the cortical laminae – such that superficial pyramidal cells pass ascending prediction errors to higher hierarchical levels and receive descending predictions from deep pyramidal cells (Mumford, 1992). In this setting, precision is thought to be encoded by the postsynaptic gain of cells reporting prediction error; i.e., the gain of pyramidal cells sending forward connections in the brain (Feldman and Friston, 2010). This is important, because many psychopathologies implicate neuromodulation and a putative failure of postsynaptic gain control. In our previous paper (Adams et al., 2012), we exploited this link to simulate the failures of active inference during SPEM that are typical of schizophrenia – whose pathophysiology is thought to involve abnormalities of dopaminergic and NMDA receptor function (Laruelle et al., 2003).

## 4 Conclusion

In summary, we have described a procedure to estimate (subjective) Bayesian beliefs that underlie oculomotor pursuit movements – using an occlusion paradigm and dynamic causal modelling. The beliefs in question here are formal Bayesian beliefs expressed in terms of normative models of oculomotor pursuit. Although this work is primarily a proof of principle – that it is possible to estimate beliefs from non-invasive eye tracking data – its results speak to the crucial role of precision or confidence in nuancing the way that we sample our world (Warren et al., 2012; Yang et al., 2012; Bogadhi et al., 2013). Indeed, it was this aspect of perceptual inference that we were interested in because–in the setting of predictive coding – the suboptimal encoding of precision or uncertainty may underlie false inference in several neuropsychiatric syndromes (Adams et al., 2013b).

From a technical point of view, this work introduces the application of dynamic causal modelling to eye movement data. In particular, it suggests that interesting questions can be addressed to response averages – in exactly the same way that event related potentials summarise average electrophysiological responses to well-controlled experimental stimuli. In terms of modelling, we have also shown that it is possible to use empirical data to inform (invert) relatively sophisticated Bayesian or normative models of behaviour. There are many carefully constructed and validated descriptive SPEM models in the literature (e.g. Barnes, 2008; Deno et al., 1995; Krauzlis and Lisberger, 1989; Krauzlis, 2004; Lisberger, 2010; Robinson et al., 1986; Shibata et al., 2005): however, the generative model that we used is distinguished in the sense that it is a special case of generic (predictive coding) models that conform to normative (Bayesian) principles. We have previously shown that formally similar generative models can reproduce both control and schizophrenic subjects’ pursuit of targets whose occlusion is either expected or unexpected, and of targets that unpredictably change direction (Adams et al., 2012). They can also reproduce the effects of contrast (sensory precision) on pursuit, such that perceived lag increases with contrast but true lag decreases, and the anticipatory initiation of pursuit of a hemi-periodic target (Perrinet et al., 2014). More generally, there are a whole series of publications using the this active inference framework to study saccadic eye movements, perceptual categorisation, omission related responses, handwriting recognition, the mismatch negativity, sequential choice behaviour and so on.

The idea of precision-weighted prediction error has important commonalities with a ubiquitous construct in SPEM modelling: that of pursuit velocity being driven by gain control of the mismatch between eye and target velocity (Barnes, 2008; Churchland and Lisberger, 2002). Indeed, Orban de Xivry et al. (2013) demonstrated that two Kalman filters (using precision-weighted prediction errors) can account for both visually guided and predictive eye movements respectively. One fundamental difference between our model and others is that our model uses predictive coding rather than optimal control, and therefore does not require efference copies of motor commands–because predictions of eye and target dynamics (i.e., corollary discharge) are generated directly by the forward model (Friston, 2011). In other words, the purpose of efference copy in optimal control is to create corollary discharge – i.e. predictions in sensory coordinates – but in predictive coding these predictions are generated directly, so efference copy itself is redundant. The cortical oculomotor system can therefore operate entirely in visual, rather than motor, coordinates (Lee et al., 2013).

Another key difference is the explicit parameterisation of hierarchical precision: in effect, the (attentional) gain control of prediction error at *every* level of the cortical hierarchy, not just at the level of eye movement generation. Having said this, it would be entirely possible to compare the evidence of different generative models based upon data of the sort analysed in this paper using DCM and response averages. Here, we have focused on comparing models with and without changes in precision; however, in principle, one can compare any model (of the same data) using Bayesian model comparison.

From a neurobiological perspective, the results reported above provide an important motivation for looking for the neuronal correlates of precision updates in electrophysiological responses. In particular, the changes in precision induced by changes in target motion noise should – under predictive coding models of oculomotor pursuit – be mediated by changes in the gain of superficial pyramidal cells in the early visual and oculomotor system. Dynamic causal modelling of event related potentials has already been used to quantify these gain changes – in terms of neural mass models and recurrent self-inhibition – using manipulations of visual precision in terms of luminance contrast (Brown and Friston, 2012). We hope to use a similar approach to assess changes in recurrent (intrinsic) connectivity using the occlusion paradigm described above and magnetoencephalography.

Clearly, it is difficult to model the physiological details of predictive coding; however, recent efforts to refine neurophysiological models of canonical microcircuitry and hierarchical (extrinsic) connections have tried to bring the underlying neuronal architectures closer to those that would support predictive coding (Bastos et al., 2012). In future work, we will use the results of the current study to guide searches of neurophysiological models that explain average electrophysiological responses to visual occlusion during oculomotor pursuit. This represents a further step in validating non-invasive measures of neuromodulatory gain control – engaged during perceptual inference – that can be used in conditions like schizophrenia.

## Figures and Tables

**Table 1:** Prior expectations of model parameters and log precisions.

Parameter class	Model parameter	Short description	Prior expectation
Kinetic	$\left(\theta_{1},\theta_{4}\right)$	Parameters encoding how gaze is attracted to the invisible location – occluder independent and dependent.	$\left(\frac{1}{4},\frac{1}{32}\right)$
	$\left(\theta_{3},\theta_{5}\right)$	Parameters encoding how gaze is attracted to the target location – occluder independent and dependent	$\left(\frac{1}{2},\frac{1}{32}\right)$
	$\left(\theta_{2},\theta_{6}\right)$	Parameters encoding the viscosity of eye and target motion (fixed between experimental conditions)	$\left(\frac{1}{2},\frac{1}{4}\right)$

Precision	$\text{ln}\;\Pi_{s}$	Log precision of sensory noise	4
	$\text{ln}\;\Pi_{x}$	Log precision of eye and target motion	4
	$\text{ln}\;\Pi_{v}$	Log precision encoding the motion of the attracting location	4

Prior	$\left(\theta_{7},\theta_{8}\right)$	Parameters encoding the amplitude and phase lag behind the invisible attracting location	$\left(1,\frac{2\pi }{32}\right)$
